# Identification of NS5B resistance-associated mutations in hepatitis C virus circulating in treatment-naïve Cameroonian patients

**DOI:** 10.1016/j.ijregi.2025.100816

**Published:** 2025-11-29

**Authors:** Aristide Mounchili-Njifon, Abdou Fatawou Modiyinji, Pretty Rosereine Mbouyap, Chavely Gwladys Monamele, Moise Henri Moumbeket-Yifomnjou, Philipe Herman Njitoyap Mfombouot, Gisele Liliane Machuetum, Pascal Ibrahim Toueyem, Simon Frederic Lissock, Paul Alain Tagnouokam-Ngoupo, Jean Paul Assam Assam, Richard Njouom

**Affiliations:** 1 Department of Virology, Centre Pasteur of Cameroon, Yaoundé, Cameroon; 2 Department of Microbiology, Faculty of Sciences University of Yaounde I, Yaoundé, Cameroon

**Keywords:** NS5B protein, Hepatitis C virus, Resistance-associated, Mutations, Cameroon

## Abstract

**Objectives:**

NS5B polymerase inhibitors are essential in the treatment of hepatitis C virus (HCV) infection. Although direct-acting antivirals (DAAs) are generally effective, their efficacy can be compromised by resistance mutations, particularly in the NS5B protein. This research aimed to identify naturally occurring mutations in the NS5B gene linked to DAA resistance in treatment-naïve Cameroonian patients with chronic hepatitis C.

**Methods:**

Whole blood samples were collected from patients with chronic hepatitis C, from which plasma was subsequently separated and stored at –80°C for molecular analysis. The NS5B gene fragments were amplified using designated primers, and nucleotide sequences were acquired via the Sanger sequencing platform.

**Results:**

Analysis of sequences revealed three genotypes: genotype 4 (38.49%), genotype 1 (38.38%), and genotype 2 (23.14%). The most prevalent subtypes were 4f (22.05%) and 1e (17.84%). The clinically significant S282T mutation, which confers high-level resistance to sofosbuvir, was detected in one patient infected with HCV genotype 1e. Similarly, the C316N substitution, associated with reduced susceptibility to non-nucleoside NS5B inhibitors, was identified in 16 patients, all belonging to genotype 1e. The Q309R mutation was detected in 19 genotype 1 sequences, and the L320F mutation was found in one genotype 4f sequence.

**Conclusions:**

Our investigation revealed that HCV patients who had not previously received DAA therapy exhibited a variety of NS5B gene alterations. Consequently, future treatment failure may be more likely due to these alterations.

## Highlights

• NS5B polymerase inhibitors form the basis of current treatment of hepatitis C virus (HCV) infection.
• The resistance profile was analyzed using Geno2pheno (hcv) 0.92 webtool.
• The mutation S282T, which confers high resistance to sofosbuvir, was found in one patient.
• The C316N mutation associated with HCV resistance has been detected.
• The Q309R mutation associated with ribavirin resistance was also detected.

## Introduction

The hepatitis C virus (HCV) is a major public health problem with severe clinical consequences worldwide [1]. It is estimated that 50 million people are chronically infected with the HCV, and approximately 10 million new infections occur each year [2]. Since 2011, there has been a significant advancement in HCV treatment with the development of direct-acting antivirals (DAAs), which can achieve a sustained virological response [3]. These DAAs developed for the treatment of HCV target the NS3/4A protease, the NS5A protein, and the NS5B polymerase, which are viral proteins involved in HCV replication [4]. NS5B polymerase inhibitors are the cornerstone of current treatment for HCV infection because the NS5B region of the hepatitis C genome is essential for viral replication, encoding the RNA-dependent RNA polymerase [5]. Despite the efficacy of DAAs with a sustained virological response rate of over 90% [6], approximately 5-10% of HCV-infected individuals fail DAA treatment. This failure results from the rapid replication rate of the HCV life cycle, low polymerase fidelity, immune system pressure, and selective drug treatment, leading to the selection and emergence of drug-resistant variants within the HCV-infected population [7].

HCV is a member of the family *Flaviviridae*, further classified as the prototypical member of the genus *Hepacivirus* [8]. It has a single-stranded positive-sense RNA genome of approximately 9600 nucleotides, encoding a single polyprotein that is further processed by viral and cellular proteases into three structural proteins (core and envelope glycoproteins E1 and E2) and seven non-structural proteins (p7, NS2, NS3, NS4A and B, and NS5A and B) [9]. Due to its high genetic heterogeneity, genomic sequencing has revealed the presence of eight HCV genotypes (1-8) and 105 subtypes that differ in nucleotide sequence by 30% and 15%, respectively [10].

Based on experience with other chronic viral infections, HCV antiviral medication resistance has emerged as a public health concern in the diagnosis and treatment of individuals with chronic hepatitis C. The characteristics of distinct resistance mutations have been widely established, based on previous clinical and laboratory evidence [11]. NS5B nucleotide inhibitors are associated with high resistance barriers [12]. The presence of resistance-associated variants in treatment-naïve patients has been reported in several countries [5,[13], [14], [15], [16], [17], [18], [19], [20], [21]]. Moreover, such studies of natural resistance mutations in treatment-naïve HCV patients may be of great importance [11,22]. However, the presence of resistance-associated mutations, particularly at the NS5B polymerase level, can reduce the efficacy of DAAs [17].

Few studies have been conducted in Cameroon to measure the frequency of mutations associated with resistance to HCV antiviral treatment. However, there are a limited reports on natural or treatment-naïve mutations. This makes the current study important [23,24], as resistance-associated substitutions (RAS) can occur naturally in HCV-infected patients prior to the initiation of DAA therapy [25]. Therefore, there is an increasing need to improve treatment efficacy in individuals treated with different regimens for each infectious genotype, especially with the presence of natural HCV RAS in DAA-naïve patients [17]. Therefore, the purpose of this study was to look at whether Cameroonian patients who had not taken DAA had primary drug resistance mutations in the NS5B area of HCV. Furthermore, these data will aid in the better selection of suitable DAA regimens for upcoming HCV control and elimination initiatives involving the Cameroonian populace.

## Methods

### Research methodology

Between January 2013 and October 2023, a total of 1728 plasma samples from treatment-naïve HCV-RNA-positive patients were received at the Centre Pasteur du Cameroun (CPC). Among them, 925 (53.5%) samples were successfully amplified and sequenced in the NS5B region and were included in the final analysis. Blood samples were collected from treatment-naïve HCV-infected patients at the CPC as part of a nationwide retrospective cross-sectional descriptive study. The CPC is the national HCV control program's focal point and Cameroon's reference laboratory for a number of diseases, including viral hepatitis. HCV patients are frequently sent to the CPC for viral load and genotyping as part of this duty. There was no further testing done; the data shown here were gathered as part of standard HCV diagnostic procedures. This was a retrospective cross-sectional study including all treatment-naïve patients with detectable HCV RNA referred to the CPC for viral load measurement and genotyping over a 10-year period (2013-2023). No patient had ever received DAAs or interferon-based therapy. Genotyping was carried out through sequencing the NS5B region of the HCV genome. As directed by the manufacturer, we measured the HCV viral load using the Abbott Real-Time HCV assay and Abbott m2000 platforms (Abbott Molecular, Wiesbaden, Germany). Briefly, the procedure involves using the Abbott m2000sp to extract RNA from 0.5 ml plasma (separated from EDTA tubes), followed by amplification on the m2000rt at a detection limit of 12 IU/ml.

### Amplification of the NS5B region

Amplification, sequencing, and phylogenetic analysis of the 382 nucleotide sequences of the NS5B genomic region were used to carry out HCV genotyping and subtyping. In summary, a QIAamp Viral RNA mini kit was used to extract viral RNA from 140 µl of plasma from HCV-positive patients who had a detectable viral load, in accordance with the manufacturer's instructions (Qiagen, Courtaboeuf, France).

The reverse transcription polymerase chain reaction (RT-PCR) step was performed using a semi-nested RT-PCR targeting the 925 nucleotides of the NS5B region. A first RT-PCR was performed using the SuperScript™ III One-Step RT-PCR System with Platinum Taq (Invitrogen, Carlsbad, USA), Pr3 (5**′**-TATGAYACCCGCTGYTTTGCTC-3**′**), and Pr4 (5**′**-GCNGARTAYCTVGTCATAGCCTC-3**′**) as primers. Amplification started with cDNA synthesis at 50°C for 30 minutes, followed by five cycles at 93°C for 30 seconds, 60°C for 45 seconds, and 72°C for 1 minute. This was immediately followed by 35 cycles at 93°C for 30 seconds, 60°C with a drop of -0.3°C between each cycle, and an extension at 72°C for 1 minute. The final extension was at 72°C for 5 minutes. The second amplification step (semi-nested PCR) targeted 382 nucleotides of the NS5B region according to the protocol developed by Sandres-Sauné et al. [26]. The reaction mixture consisted of 5 µl of 10X buffer, 3.5 µl MgCl_2_, 1 µl dNTPs, 0.4 µM each of the inner primers Pr3 (forward) and Pr5 (reverse: 5**′**-GCTAGTCATAGCCTCCGT-3**′**), and 0.25 µl of Taq polymerase enzyme. To the total reaction mixture, 2 µl of the first RT-PCR products were added. The thermal cycling conditions were as follows: one cycle at 95°C for 5 minutes, then 35 cycles at 95°C for 30 seconds, 55°C for 30 seconds, 72°C for 30 seconds, and a final extension at 72°C for 10 minutes. The expected amplicon size for the nested product was approximately 382 bp was visualized on 2% agarose gel electrophoresis.

### Sequencing and phylogenetic analyses

Using the Genome Lab DTCS-Quick Start kit, all nested PCR products of the NS5B region were sequenced using the Sanger technique (Beckman Coulter, Paris, France) in compliance with the guidelines provided by the manufacturer. Using the CLC Main Workbench software (version 5.5.2), forward and reverse sequences were manually edited before consensus sequences were produced. The HCVnet genotyping tool (https://www.genomedetective.com/app/typingtool/hcv/) was used to assign the HCV genotype and subtype, and the tree was inferred using MEGA version 11 under the General Time Reversible model with gamma-distributed rate variation and a proportion of invariant sites (GTR + Γ + I), selected as the best-fitting model by jModelTest 2 according to the Bayesian information criterion. Branch support was assessed with 1000 bootstrap replicates; only bootstrap values ≥70% are displayed.

### Study of resistance-associated mutations in the HCV NS5B polymerase

To detect the various natural substitutions and assess their impact on resistance, all 925 NS5B sequences (from genotypes 1, 2, and 4) were subjected to Geno2pheno (hcv) 0.92, which provides a list of mutations and predictions of phenotypic resistance to antiviral drugs for each strain [3].

## Results

### Demographic characteristics

#### Distribution and frequency of NS5B resistance-associated variants’ genotype (subtype)

The study included 925 consecutive patients who satisfied the eligibility requirements, of whom 536 (57.95%) were women and 389 (42.05%) were men, resulting in a M/F sex ratio of 0.73. With a mean of 68.02 and a median of 70 years, the age ranged from 5 to 96 years.

**Figure 1:**
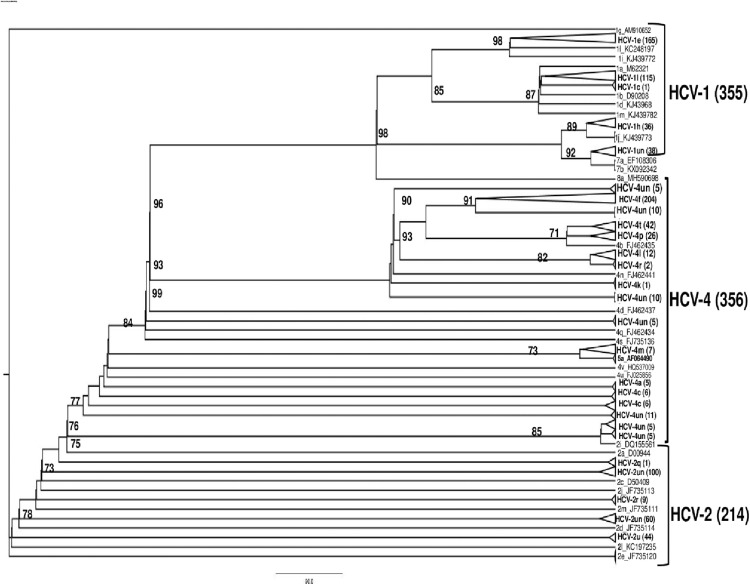
Phylogenetic analysis based on partial NS5b gene sequences of Cameroonian HCV isolates and reference strains. The phylogenetic tree was based on 925 partial sequences of the HCV NS5b gene from samples collected in Cameroon between 2013 and 2023 and reference strains, which are identified by their accession number. The virus sequences described here are indicated in bold and grouped in the corresponding clusters. The tree was constructed by the maximum likelihood method using the Kimura two-parameter model. The reliability of tree topologies was estimated by bootstrap analysis with 1000 pseudo-replicated datasets, and for clarity, bootstrap values below 70 are not shown. All 925 sequences have been submitted to the GenBank database with individual access numbers. HCV, hepatitis C virus.

Three genotypes were identified by sequencing the NS5B region in 925 blood samples from HCV-positive individuals: genotype 4 was the most common (38.49%), followed by genotype 1 (38.38%) and genotype 2 (23.14%). The predominant subtypes were 4f (22.05%) and 1e (17.84%) (see Table 1 and Figure 1 for complete genotype/subtype distribution).

**Table 1:** Frequency of amino acid substitutions in the NS5B region of hepatitis C virus in 925 treatment-naïve Cameroonian patients, stratified by genotype and subtype.

Genotypes (%)	Subtypes (n)	Mutations	N	%
**1 (38, 38%)**	1e (n = 165)	**K270R**	50	5.41%
		**S282T**	1	0.11%
		**Q309R**	19	2.05%
		**D310N**	10	1.08%
		**C316N**	16	1.73%
		**V321I**	2	0.22%
		**A333V**	12	1.30%
	1l (n = 115)	**K270R**	4	0.43%
		**C316H**	1	0.11%
		**V321I**	3	0.32%
		**A333V**	28	3.03%
	1h (n = 36)	**M300T**	5	0.54%
	1un (n = 38)	**A300S**	1	0.11%
		**T300S**	23	2.49%
**2 (23, 14%)**	2un (n = 204)	**E237G**	3	0.32%
		**L241Q**	52	5.62%
		**K270R**	3	0.32%
		**M289L**	10	1.08%
		**D310N**	64	6.92%
		**N310D**	4	0.43%
		**V321I**	1	0.11%
		**A329T**	16	1.73%
		**A329V**	7	0.76%
**4 (38, 49%)**	4f (n = 204)	**E237G**	45	4.86%
		**K270R**	182	19.68%
		**S282L**	1	0.11%
		**N310D**	139	15.03%
		**N316C**	71	7.68%
		**L320F**	1	0.11%
		**V321I**	3	0.32%
		**D330E**	126	13.62%
		**D330Q**	10	1.08%
		**R333F**	4	0.43%
	4t (n = 42)	**D310N**	5	0.54%
		**G330K**	13	1.41%
	4p (n = 26)	**V329I**	2	0.22%
		**D330E**	3	0.32%
		**D330Q**	1	0.11%
		**R333K**	26	2.81%
	4r (n = 2)	**C316H**	2	0.22%
		**K333R**	2	0.22%
	4l (n = 12)	**M300T**	10	1.08%
		**G333A**	2	0.22%
	4a (n = 5)	**P300T**	2	0.22%
		**P300S**	5	0.54%
	4m (n = 7)	**T300S**	2	0.22%
		**V300I**	1	0.11%
		**K333R**	3	0.32%

Un: unclassified.

The HCV nucleotide sequences described in this study have been submitted to the Genbank database under the following accession numbers: OR477026-OR477034, OR477036, OR477038-OR477046, OR477048-OR477046, OR477048-OR477054, OR477056- OR477061, OR477063-OR477067, OR477069-OR477071, OR478291-OR478378, OR480599-OR480642, OR480646, OR490539-OR490588, OR520331, OR520333, OR520337, OR520340, OR520345-OR520374, OR765771-OR765774, OR765776-OR765810, OR765820, OR765823- OR765824, OR765826- OR765830, OR818228-OR818286, OR818288-OR818301, OR818302, OR818307, OR818312-OR818322, OR818329-OR818336, OR818341-OR818349, OR825140, OR825144, OR825146-OR825180, OR825183, OR825184-OR825305, OR825307-OR825321, OR837048, OR837051-OR837053, OR902261, OR902264-OR902271, OR923596, OR923662, OR923663-OR923680, OR923682-OR923692, OR923694, OR923696-OR923700, OR923704, OR923706-OR923713, PP484706-PP484712, PP484714-PP484746, PP484748, PP484750- PP484800, PP484802- PP484862, PP484864, PP484866- PP484867, PP484869, PP484871-PP484877, PP583866- PP583875, PP583877-PP583908, PP583910- PP583967, PP583969- PP584014.

#### Frequency of mutations associated with natural NS5B resistance

In genotypes 1e, 2, and 4f, natural NS5B RASs were prevalent (Table 2), with a significant frequency of S282T (1/165, 0.61%), Q309R (11.52%), D310N (6.1%), C316N (9.7%), and V321I (1.21%) in genotype 1e. In addition, several natural RAS were detected at different frequencies: E237G (45/204, 22.06%), S282L (0.49%), N310D (68.14%), N316C (34.80%), L320F (0.61%), and V321I (1.47%) in HCV genotype 4f. Four mutations were detected in genotype 2 at positions E237G, M289L, D310N, and V321I (Table 1).

**Table 2:** Clinically relevant NS5B RAS detected in treatment-naïve Cameroonian patients with chronic hepatitis C virus infection.

RAS occupying NS5B positions	Inhibitors	Level of resistance	Genotype	Treatment regimen	Number of sequences (%)
E237G	Sofosbuvir	Reduced susceptibility	2 and 4f	SOF/VEL, SOF/LED	48 (5.19%)
S282T	Sofosbuvir	High resistance to sofosbuvir	1e	SOF/VEL	1 (0.11%)
M289L	Sofosbuvir	Reduced susceptibility	2	SOF/VEL	10 (1.08%)
L320F	Sofosbuvir	Sofosbuvir resistance	4f	SOF/VEL	1 (0.11%)
V321I	Sofosbuvir	Reduced susceptibility	1e; 1l; 2 and 4f	SOF/VEL	7 (0.76%)
Q309R	Ribavirin	Reduced susceptibility	1e	SOF/VEL, SOF/VEL/RIB, SOF/LED/RIB	19 (2.05%)
D310N	Ribavirin	Reduced susceptibility	1e; 2 and 4t	SOF/VEL	79 (8.54%)
C316N	Sofosbuvir	Sofosbuvir resistance	1e	SOF/VEL	16 (1.73%)
S282L + V321I	Sofosbuvir	Reduced susceptibility	4f	SOF/VEL	1 (0.11%)

RAS, resistance-associated substitutions; SOF/LED, sofosbuvir/ledipasvir; SOF/VEL, sofosbuvir/velpatasvir; SOF/VEL/RIB, sofosbuvir/velpatasvir/ribavirin.

In different parts of the genome, resistance mutations and RAS arise naturally because the viral polymerase lacks the correcting exonuclease function. These mutations can occur throughout the viral genome, including NS3, NS5A, and NS5B [27]. These mutations have treatment implications by affecting the sensitivity of antiviral molecules. Among 925 patients, single NS5B inhibitor-resistant mutations were identified as spontaneous NS5B RAS, including E237G (5.19%), S282T (0.11%), M289L (1.08%), Q309R (2.05%), D310N (8.54%), C316N (1.73%), L320F (0.11%), V321I (0.76%), and V329I (0.43%). These frequencies represent the proportion within the total cohort (N = 925) (Table 2). Among the 98 strains with ribavirin resistance associated with a single mutation, the HCV isolates with the Q309R and D310N mutations were 19/925 and 79/925, respectively. The S282T mutation, known to be highly resistant to sofosbuvir, was found in one HCV isolate, whereas the C316N mutation, known to be resistant to sofosbuvir, was found in 16/925 HCV isolates. The M289L and L320F mutations, known to confer reduced sensitivity to sofosbuvir, were present in 10/925 and 1/925 of the isolates in our study, respectively. Furthermore, one HCV strain exhibited a double mutation S282L + V321I in the NS5B region, which is known to confer high-level resistance to sofosbuvir. This combination was identified in a patient infected with genotype 4f and warrants particular attention due to its potential to cause treatment failure. All NS5B RAS common to DAAs were linked to ribavirin resistance in genotype 1e in our sample of patients at locations L159, N244I, T329I, and A333E (lower HCV sensitivity to ribavirin), with mutations detected in 98 HCV-infected patients. In patients with genotype 1a (14/16, 87.5%), simultaneous detection of RAS NS5B was more common than in patients with genotype 6a (2/16, 12.5%). Multiple NS5B RAS were identified in genotypes 1e and 4f, with genotype 4r containing S282R + V321I and genotype 1e, including E237G + S282R + Q309R + V321I.

## Discussion

One of the main causes of liver cirrhosis, hepatocellular cancer, and death is chronic HCV infection. In addition, it is estimated that approximately 40-70% of patients develop non-hepatic alterations during chronic infection [28]. Although interferon-free DAA therapy has been a major development in the treatment of HCV, the persistence of the virus under drug pressure and the persistence of a natural polymorphism that may correlate with DAA resistance are considered major challenges to the success of HCV therapy [5]. NS5B is the main target of DAAs, which directly prevent viral multiplication; several mutations that lessen the effectiveness of NS5B inhibitors have been documented and might be innate in patients who have not received treatment. These mutations arise due to the absence of the corrective exonuclease activity of the viral polymerase [16]. The incidence of NS5B mutations in HCV genotype 1, 2, and 4 patients in Cameroon who are new to DAA therapy is reported in this study.

Our study, which we believe is one of the few to have estimated natural NS5B RASs in DAA-naïve HCV patients in Cameroon to provide optimal treatment, was carried out in 925 treatment-naïve patients who were successfully amplified for the NS5B fragments under investigation. This is because Cameroonian data on HCV drug resistance is extremely limited.

Concerning the NS5B gene, a global analysis revealed that NS5B DAA resistance substitutions are rare [29]. Relevant natural amino acid polymorphisms were found in genotypes 1e, 1l, 2, 4f, 4t, 4p, and 4l in our investigation. Several studies have demonstrated the occurrence of natural mutations in the NS5B region. Two studies carried out in Cameroon, the first on 252 treatment-naïve patients in the NS5B region in 2016, had already presented several natural mutations, indicating the importance of monitoring and tracking resistance-associated mutations [24], whereas the other study on 190 HCV RNA-positive patients revealed the presence of a resistance-associated mutation in the NS5B region [23]. In addition, a study carried out in Egypt on 27 treatment-naïve HCV-infected patients and eight non-responders, multiple resistance mutations in the NS5B region were detected in several patients [5]. A study in South Africa on 42 HCV-infected and treatment-naïve patients also revealed the presence of multiple resistance mutations in the NS5B region [15]. Furthermore, in a study of 108 HCV-1 patients in Argentina, resistance mutations in the NS5B region were detected in 6.3% of these patients [30].

The significant S282T mutation confers a high level of sofosbuvir resistance despite the low frequency of this mutation in the HCV NS5B region [24]. This S282T mutation was detected in our study in a patient with genotype 1e, consistent with several previous studies showing the low frequency of the S282T mutation in the NS5B region but inducing a high level of sofosbuvir resistance. A study carried out in 2024 in Egypt confirmed two cases of S282T mutations [31]; similarly, a study in Pakistan equally identified two cases of S282T mutations [21]. In addition, several previous studies have shown that patients who did not respond well to sofosbuvir-based treatment regimens had an S282T mutation [32,33]. However, several other studies in Cameroon, Africa, and even Europe did not detect the S282T mutation. This explains the low prevalence of this mutation in HCV patients [5,24,34].

The C316N mutation, including sofosbuvir, has been reported to confer resistance to DAA therapy [35]. This study showed that treatment-naïve HCV patients in Cameroon had mutations that conferred resistance to HCV NS5B polymerase, i.e., genotype 1e, in 16 patients. Our results are in line with a study from Morocco showing that the C316N mutation was more prevalent in genotype 1 in DAA treatment-naïve patients [16]. A study conducted in Asia had also shown the high frequency of C316N mutations in treatment-naïve genotype 1b patients with chronic hepatitis C [20].

Mutations at locations Q309R, D244N, and A333E are linked to ribavirin resistance.

Our investigation revealed that 19 HCV patients who had not received therapy had a frequency of 2.05% Q309R mutations. Our findings are in line with recent research demonstrating the existence of numerous Q309R mutations in patients who have not received therapy, such as an Egyptian study that found that out of 27 drug-naïve HCV infections, the Q309R mutation had a frequency of 5.8% [5]. Another study in Brazil reported that among 69 drug-naïve individuals infected with HCV, the most common mutation was Q309R (29%) [36]. Nevertheless, our investigation did not find the D244N mutation, in agreement with a study conducted in Egypt in 2021, where this mutation was also not detected [5]. Conversely, the mutation at position 333 was detected in our study; however, with a different protein alteration, i.e., A333V, with a frequency of 4.32%.

Our study reported E237G mutations in two HCV strains, GT-4f and GT-2, from our HCV-naïve samples. This finding agrees to some extent with an Egyptian study of 27 treatment-naïve patients that found an E237G mutation in the NS5B region in the only non-susceptible GT-4o HCV strain, and E237G/A mutations in four other strains [5]. In another study of 333 treatment-experienced patients, 10 individuals experienced virological relapse, and at the time of relapse, two patients with genotypes 1a and 4d had an E237G mutation found in them (Manns *et al*., 2016). Another study found the presence of an E237G substitution in a GT-4 patient without a significant therapeutic response [10].

The L320F polymerase mutation has been observed to confer low resistance to HCV polymerase inhibitors such as sofosbuvir *in vivo* [37]. Our study reported the presence of this mutation at L320F in a patient infected with HCV genotype 4f. These results are consistent with a study carried out in Brazil, where an L320F mutation was also identified in a treatment-naïve HCV-infected patient [13]. In addition, a study has shown that the M289L mutation reduces sensitivity to sofosbuvir by a factor of 2-20 [38], whereas our study reported the presence of this M289L mutation in 10 patients infected with HCV genotype 2.

It is still unknown how HCV resistance mutations affect the virus's capacity for *in vivo* replication. Therefore, more research is required to fully evaluate how each variation affects the degree of resistance or susceptibility to HCV medications. As a result, developing an HCV vaccine is not only important but also essential for the long-term management of HCV infection. Nowadays, DAA-based treatment plans are effective in curing almost all chronic HCV infections. Nonetheless, considering the astronomically high global infection rate and the continuous identification of novel HCV subtypes, some of which harbor pre-existing resistance mutations, the rise of multidrug-resistant viruses continues to be a serious worry.

The present study was limited to a single HCV region, NS5B, and only treatment-naïve, chronically infected patients were included. Future studies should focus on the prevalence of innate HCV antiviral resistance, as well as resistance acquired during HCV treatment. Therefore, the creation of an HCV vaccine is not only essential but also a priority for the future management of HCV infection. The results of this study could help determine which DAA treatment plan is optimal for HCV patients.

## Conclusion

Our research revealed that a number of mutations in the NS5B genes under analysis may raise the chance of treatment failure in Cameroonian HCV patients receiving regimens containing DAA. The high virological success rate of sofosbuvir-based regimens, the uncommon occurrence of RAS in non-responder patients, the lack of data developed for RAS in genotype 4 patients compared with other genotypes, and the overall difficulty of completely understanding the impact of some of these substitutions may all be factors linked to treatment failure in Cameroon. To investigate the link between the alterations found in our study and HCV resistance, more research is required.

## Declaration of competing interest

The authors have no competing interests to declare.
